# Fusion of Ubiquitin to HIV Gag Impairs Human Monocyte-Derived Dendritic Cell Maturation and Reduces Ability to Induce Gag T Cell Responses

**DOI:** 10.1371/journal.pone.0088327

**Published:** 2014-02-05

**Authors:** Shanthi Herath, Adel Benlahrech, Timos Papagatsias, Takis Athanasopoulos, Zineb Bouzeboudjen, Catherine Hervouet, Linda Klavinskis, Andrea Meiser, Peter Kelleher, George Dickson, Steven Patterson

**Affiliations:** 1 Department of Immunology, Imperial College London, Chelsea and Westminster Hospital, London, United Kingdom; 2 School of Biological Science, Royal Holloway University of London, Egham, Surrey, United Kingdom; 3 Peter Gorer Department of Immunobiology, Guys Hospital, Kings College London, London, United Kingdom; Ragon Institute of MGH, MIT and Harvard, United States of America

## Abstract

The efficient induction of CD8 T cell immunity is dependent on the processing and presentation of antigen on MHC class I molecules by professional antigen presenting cells (APC). To develop an improved T cell vaccine for HIV we investigated whether fusing the ubiquitin gene to the N terminus of the HIV gag gene enhanced targeting to the proteasome resulting in better CD8 T cell responses. Human monocyte derived dendritic cells (moDC), transduced with adenovirus vectors carrying either ubiquitinated or non-ubiquitinated gag transgene constructs, were co-cultured with autologous naïve T cells and T cell responses were measured after several weekly cycles of stimulation. Despite targeting of the ubiquitin gag transgene protein to the proteasome, ubiquitination did not increase CD8 T cell immune responses and in some cases diminished responses to gag peptides. There were no marked differences in cytokines produced from ubiquitinated and non-ubiquitinated gag stimulated cultures or in the expression of inhibitory molecules on expanded T cells. However, the ability of moDC transduced with ubiquitinated gag gene to upregulate co-stimulatory molecules was reduced, whilst no difference in moDC maturation was observed with a control ubiquitinated and non-ubiquitinated MART gene. Furthermore moDC transduced with ubiquitinated gag produced more IL-10 than transduction with unmodified gag. Thus failure of gag ubiquitination to enhance CD8 responses may be caused by suppression of moDC maturation. These results indicate that when designing a successful vaccine strategy to target a particular cell population, attention must also be given to the effect of the vaccine on APCs.

## Introduction

The urgency for a prophylactic HIV vaccine is evident by the sustained global increase in HIV-1 infection. Despite the best efforts of over 20 years of research an effective HIV vaccine remains elusive, compounded by the diversity of the virus and its ability to evade immune responses. Although HIV-1 infection induces a strong antibody response, many of the antibodies are unable to neutralise the broad range of strains that now exist [1]. Recent progress has been in isolating broadly neutralising antibodies from HIV infected individuals. However such antibodies only appear after infection for several years with high virus loads and require very extensive changes in germ line immunoglobulin changes which will be difficult to achieve with vaccinations [2]. Nevertheless, induction of effective neutralising antibodies remains at the forefront of developing a preventative vaccine that provides long term immunity. However, promising studies have implicated the importance of CD8 T cells in controlling HIV replication, and current research is now focusing on targeting CD8 T cells to develop a sterilising vaccine [3]–[8]. In chronically HIV-infected individuals CD8 T cells are found to respond to a variety of HIV proteins and it has been shown that responses to the gag protein, a more highly conserved HIV protein, correlated with reduced viral loads, with an increase in breadth of gag responses appearing to further enhance control of viral replication [5]. Thus current studies aim to develop vaccines that recognise multiple CD8 T cell epitopes to HIV proteins and previous work has shown that modifying the gag protein increased CTL responses [9].

Although antigenic proteins usually contain numerous potential CD8 epitopes there is an immunodominant hierarchy such that in acute infections or vaccination there are responses to only one or two epitopes [10]. Consequently, in HIV, the T cell response is characterised by restricted breadth, usually one of two epitopes [11] in the acute phase which broadens at the chronic stage [12], [13]. Generation of cytotoxic T cells (CTL) is dependent on the presentation of peptides bound to the MHC class I molecules on the surface of antigen presenting cells and levels of MHC class I peptide complexes is a factor in determining the immunodominant hierarchy [10]. The proteasome is the main proteolytic organelle in the generation of MHC class I peptides [14] and proteins are targeted to the proteasome for degradation through a process of ubiquitination where ubiquitin is added to lysine residues by a series of ligases [15], [16]. Ubiquitination thus plays a key role in MHC class I presentation [17] and it has been previously shown that ubiquitinating a transgene enhances Class I presentation and consequently CD8 T cell responses and observed when the transgene is delivered in an Ad5 vector or by DNA immunisation [18], [19].

Dendritic cells (DC) are the most potent antigen presenting cells also having the unique ability to effectively stimulate naïve T cells and thus are essential for the induction of primary T cell responses and targets for effective vaccines [20]. There are two main types of DC, plasmacytoid DC and myeloid DC, but the latter is thought to be primarily responsible for inducing T cell responses to infections and vaccines. There are several distinct populations of myeloid DC in both mouse and human [21] and for this study we chose to investigate our vaccine constructs using human monocyte-derived DC (moDC). In vivo moDC are regarded as inflammatory DC [22], [23] and thus may serve to augment an already initiated response. Monocyte derived DC have been used in therapeutic vaccines for cancer [24], [25] and for SIV [26] and HIV infection [27]. Based on previous work showing that CD8 T cell responses to the HIV gag protein resulted in control of viral replication, we hypothesised that targeting HIV gag to the DC proteasome would result in increased MHC class I peptide presentation and enhance the magnitude and breadth of CD8 T cell responses. We generated an Adenovirus vector containing a ubiquitinated gag transgene and explored T cell responses stimulated by transduced moDC. We find that although fusion of ubiquitin to gag promotes its proteasomal degradation, T cell responses induced by transfected moDC are reduced and may reflect impaired up-regulation of co-stimulatory molecules and increased Il-10 production caused by the ubiquitin-gag fusion vaccine.

## Materials and Methods

### Ethics Statement

Ethical approval was obtained from the Riverside Research Ethics Committee and informed written consent was obtained prior to patient blood collection.

All *in vivo* procedures were performed in accordance with United Kingdom Home Office regulations and Kings College London Ethics Committee.

### Blood Samples

Peripheral blood mononuclear cells (PBMCs) were isolated from blood leukocyte cones (National Blood Transfusion Service, UK) or from HIV-1^+^ patients attending Chelsea and Westminster hospital (London, UK), by density gradient centrifugation with Lymphoprep™ 1077 (PAA Laboratories, GmbH, UK) followed by a 50% Percoll (Sigma, UK) gradient to obtain a high-density fraction enriched in lymphocytes and a low-density fraction enriched in monocytes. Fractions were aliquoted and stored in liquid nitrogen until required.

HLA-A*0201 donors were used for the MART experiments and were determined by firstly identifying HLA-A*02 positive individuals by staining donor PBMC (National Blood Transfusion Service, UK) with an anti-HLA-A*02 antibody and then analysing by flow cytometry. Once identified, cells were stained with an APC pentamer expressing the dominant peptide to the MART protein [ELAGIGILTV, Peprotech, UK], which is recognised by HLA-A*0201 individuals, and analysed by flow cytometry. Cells from MART+ donors were then stored in liquid nitrogen until required.

### Generation of Dendritic Cells

Monocyte derived dendritic cells (moDCs) were isolated from the monocyte rich fraction by magnetic bead separation using anti-CD14 coated magnetic beads (Miltenyi Biotec). Purified monocytes were cultured in complete medium (RPMI containing 10% FBS, 100 IU/mL penicillin, 0.1 mg/mL streptomycin, and 2 mM L-glutamine; Sigma-Aldrich) supplemented with GM-CSF (100 ng/mL) and IL-4 (50 ng/mL; R&D Systems), which was replenished every other day. On day 7, immature DCs were harvested, washed twice and resuspended in serum-free RPMI-1640 prior to transduction with Ad5 vectors. Fresh moDCs were generated on a weekly basis to boost expanding T cell cultures for a minimum of 3 to 4 weeks.

### Ad5 Gene Strategy

In this work we used mammalian codon-optimized full length gag domain from HIV-1 Clade C isolate CN54, (encoding the same sequence as the gag gene in a vaccine construct used previously in the EuroVacc 02 trial [28]) and a molecular clone for gag from HIV-1 Clade C ZM96 (a kind gift from Dr Beatrice Hahn, University of Alabama). Gag-protein was expressed by E1/E3 deleted replication incompetent Adenovirus 5 vectors in two versions: 1) as Gag alone and 2) with Ubiquitin fused to the N-terminus of Gag in which the terminal Glycine of ubiquitin has been changed to Valine to prevent cleavage by Ubiquitin hydolases. The two constructs were sequence verified by GeneArt (Regensburg, Germany) and protein translation was verified by Western blotting.

### Ad5-CMV-MART-1

The MART-1 gene was a kind gift from Dr Lisa Butterfield [29] and came in a standard VR1012 vector. The MART-1 gene was cloned via pcDNA3.1(−) (*NotI* and *BmHI*) into the pShuttle-CMV (*NotI* and *HindIII*). The virus was generated using the AdEasy protocol [30] which was then purified using the Adenopure Kit (Puresyn Inc.).

### Ad5-CMV-1xUbG76VMART-1

A pcDNA3.1(+) plasmid vector carrying tetra Ubiquitin with a Glycine to Valine mutation in position 76 at the N-terminus and an HA tag at the C-terminus was used for construction of the ubiquitinated MART-1 constructs. The MART-1 gene was amplified by PCR using the VR1012-MART-1 as template. The 5′primer had an incorporated *KpnI* site and 3′primer an *EcoRI* site. These sites were used to clone the PCR-amplified gene into pcDNA3.1(+)4xUbG76V-HAtag. Three of the 4 Ubiquitin moieties were removed with *XhoI* digestion resulting in pcDNA3.1(+)1xUbG76V-MART-1-HA. 1xUbG76V-MART-1-HA was lifted from the pcDNA(+) vector and inserted into the pShuttle CMV using *SalI* and *HindIII* enzymes. The viruses were made and purified as described for Ad5-CMV-MART-1.

### Transduction of DCs with Ad5 Vectors

DCs were transduced with different Ad5 vectors at a ratio of 5000 virus particles (measured using the Quant-it PicoGreen assay kit, Invitrogen) of Ad5 virus per cell for 1 hr at 37°C. Cells were harvested and resuspended at a final concentration of 10^5^ cells/mL in culture medium (RPMI-1640 supplemented with 5% pooled human AB serum (Sigma Aldrich, UK), 1% penicillin, 1% streptomycin and 1% L-glutamine (PAA Laboratories)). Cells were incubated overnight then matured the following day with bacterial lipopolysaccharide (LPS, 1 µg/ml, Sigma Aldrich) and IFNγ (1000 U/ml, Miltenyi Biotec) for a further 24 hrs.

To assess proteasomal degradation of targeted vaccine genes, DCs were transduced with the different Ad5 vectors for 24 hrs, and following maturation were cultured in the presence or absence of a 10 µM of a proteasomal inhibitor, MG132 (Sigma-Aldrich, UK) for a further 24 hrs.

To assess the impact of transduction on the maturation of DCs, DCs were transduced with the different Ad5 vectors for 24 hrs, matured with LPS and IFNγ for a further 24 hrs and then surface stained for anti-CD80-PE (clone 2D10, BioLegend), anti-CD83-FITC (clone HB15e, BD UK), anti-CD86-APC (clone IT2.2, BioLegend), anti-HLA-DR-PECy5 (clone G46-6, BD, UK) and anti-PDL1-FITC (clone MIH2, AbD Serotec).

### Isolation of Naïve T Cells

Autologous naïve T lymphocytes were isolated from the lymphocyte rich fraction by negative selection by firstly enriching for T cells using the Pan T cell isolation kit II (Miltenyi Biotec) followed by depletion of CD45RO memory T cells using anti-CD45RO coated magnetic beads (Miltenyi Biotec) according to the manufacturer’s instructions. Purity of isolated cells was greater than 90% as determined by flow cytometry.

### Cell Culture

Purified naïve T lymphocytes were cultured with transduced moDCs in culture medium and on day 7 and every other subsequent day thereafter during culture they were supplemented with IL-2 (50 U/ml), IL-15 (2.5 ng/ml) and IL-7 (10 ng/ml). Cells were harvested weekly, counted, viability assessed by Trypan Blue (Sigma-Aldrich, UK) exclusion and resuspended at a concentration of 2×10^6^ cells/mL. They were then co-cultured with a new batch of autologous Ad5-transduced mature DCs at a ratio of 20∶1 in culture medium supplemented with the aforementioned cytokines. T cells were stimulated with transduced moDC three or four times to expand antigen specific T cells.

To assess T cell responses, cells were restimulated with peptides (NIBSC, UK) spanning the gag protein either individually or in pools as indicated in the Results, and were used at a final concentration of 1 µg/ml.

### Mice and Immunisation

C57BL/6 mice were purchased from Harlan, UK, and 6–8 week old female mice were used in experiments. Mice were immunised with 10^10^ VP of either gag construct subcutaneously as described previously [31]. All *in vivo* procedures were performed in accordance with Institutional and United Kingdom Home Office regulations for animal experimentation. Immunisations were carried out under anaesthesia using best practice.

### IFNγ ELISpot

HIV gag-specific IFNγ T cell responses were determined by restimulation of human T cells or, 7 day post injection mouse splenocytes, with gag overlapping peptides (15-mers overlapping by 11aa, NIBSC, UK) and quantified by an ELISPOT assay (anti-human IFNγ ELISPOT, Mabtech AB, Sweden or anti-mouse IFNγ ELISpot, U-CyTech, The Netherlands) according to the manufacturer’s instructions with the following modification to the human ELISpot protocol. After incubation with the secondary antibody, plates were washed and ABC peroxidase-avidin-biotin complex (Vector labs, UK) was added for 1 h at room temperature. Spots were then developed by addition of filtered AEC (Sigma Aldrich, UK) substrate solution for 4 min. Development was stopped with water and the plates were dried overnight before being read using an automated AID ELISPOT reader (AutoImmun Diagnostika, Germany). Mouse ELISpot plates were read on a Bioreader 5000 (Bio-Sys, Germany).

### Intracellular Cytokine Staining

After 3–4 weeks of T cell expansion, cells were harvested, washed twice and rested in cytokine-free culture medium for 48 hrs. Following this time, cells were washed, recounted and seeded in 96 well plates at a density of 10^6^ cells/100 µl. DC, which were transduced with the different Ad5 vectors as previously indicated and were matured overnight with LPS and IFNγ, were co-cultured with the T cells at a ratio of 1∶10. 121 individual HIV-CN54 gag overlapping peptides (15-mers overlapping by 11aa, NIBSC, UK) were pooled into 22 pools such that each pool contained 11 peptides and each peptide was present in 2 pools. Each pool was then added to wells in duplicate at a concentration of 11 µg/ml. Negative control wells consisted of cells cultured in the absence of peptides but in the presence of 0.025% dimethyl sulfoxide (DMSO, Sigma Aldrich, UK). Phorbol 12-myristate 13-acetate (PMA, 50 ng/ml, Sigma Aldrich, UK) and ionomycin-(1 µg/ml, Sigma Aldrich, UK) stimulated cells served as a positive control. Cells were incubated for 1 hr at 37°C, after which time Brefeldin A was added (10 µg/ml, Sigma Aldrich, UK) and cells were incubated for a further 5 hrs at 37°C. After this time, cells were harvested and surface stained for 20 minutes at 4°C with anti-CD8-APC H7 (clone SK1), and CD3-Percp-Cy5.5 (clone SK7), (both from BD Biosciences, UK). Cells were washed, fixed and permeabilised with the BD Cytofix/Cytoperm kit (BD Biosciences, UK) according to the manufacturer’s instruction. Following permeabilisation, cells were stained for 30 minutes at room temperature with an anti- IFNγ -PE-Cy7 antibody (clone 4S.B3, eBioscience, UK), anti-TNF-α-FITC (clone 6401.1111, BD Bioscience, UK) or anti-IL10-PE (clone JEs3-19F1, BD Bioscience, UK) or in combination. Finally, cells were washed and fixed with BD stabilising fixative (BD Biosciences, UK). Cells were acquired using a 3-laser configuration LSRII flow cytometer (BD Biosciences, USA). Cytokine secretion by antigen specific T cells were analysed by FlowJo (Tree Star Inc, USA). The gating strategy for the Identification of T cells was performed as previously published in [32].

### IL-10 ELISA

Using a standard ELISA protocol and matched antibody pairs, levels of IL-10 were determined in culture supernatants. Briefly, 96 well plates (Nunc, UK), were coated with 4 µg/ml of human anti-IL-10 monoclonal antibody (R&D Systems, UK). Following an overnight incubation at 4°C, plates were washed and blocked with 200 µL of StartingBlock™ (Thermo Scientific, UK) for 30 mins at room temperature. Blocking buffer was removed, and without washing, supernatants were added to the plates along with cytokine standards ranging from 4000 pg/mL to 62.5 pg/mL and incubated for a minimum of 2 hrs at room temperature. Plates were then washed and incubated with 0.5 µg/mL of anti-IL-10 biotinylated antibody (R&D Systems, UK) for 1 hr at room temperature. Plates were washed and incubated with 0.1 µg/mL of Strepavidin peroxidise (High Sensitivity, PIERCE, UK) for 30 mins at room temperature. Following a final wash, TMB substrate (Sigma, UK), was added and the reaction was stopped with Stop Solution (Sigma, UK). Plates were read using an ELISA plate reader at 450 nm.

### Immunogenicity Assessment of Gag Vectors in Lymphocytes from HIV-1-infected Individuals

40–60 ml of blood was collected from three aviremic HIV-1 infected patients receiving highly active anti-retroviral therapy. Ethical approval was obtained from the local Ethics Committee and written informed consent was obtained from all patients. The samples were processed as described by [33] with slight modification. In brief, PBMC were isolated and rested overnight at 4°C in complete medium supplemented with 2% Hepes (Sigma Aldrich, UK). Monocytes were positively isolated using CD14 micro-beads (Miltenyi Biotec, UK) and the remaining cells were cryopreserved at in liquid nitrogen. DC were generated from monocytes over seven days as previously indicated then transduced with either Ad5 expressing non-ubiquitinated gag (Ub^−^gag) or ubiquitinated gag (Ub^+^gag). Cells were matured with LPS and IFNγ overnight then co-cultured with autologous thawed PBMC for 7 days at a ratio of 1∶10. HIV gag-specific IFNγ T cell responses were quantified by ELISPOT assay as described above. A pre-culture period in the presence of transfected DC was used as the T cell response has been noted to wane in individuals receiving antiviral therapy [34] and we wished to detect responses to low frequency sub dominant epitopes. Cultured ELISPOTS have been shown to increase the sensitivity of detection for T cell responses [35].

### Flow Cytometry

Prior to co-culture with transduced DCs, 10^6^ T cells were harvested, washed and stained with anti-CD8-APC.H7 (clone SK1), anti-CD3-Percp-Cy5.5 (clone SK7), anti-PD-1-APC (clone MIH4), anti-CD38-FITC (cloneHIT2) (all from BD Bioscience, UK), anti-CD160-PE (clone 688327), anti-TIM3-PE (clone 344823)(both from R&D, UK) and anti-LAG3-FITC (clone 17B4, Enzo Life Sciences, UK) for 20 mins at room temperature. Following this incubation, cells were washed and fixed with BD stabilising fixative (BD Biosciences). One hundred thousand cells were acquired within a live gate using a 3-laser configuration LSRII flow cytometer (BD Biosciences, USA) and analysed by FlowJo (Tree Star Inc, USA).

To assess gag protein production, transduced DCs were harvested, washed and stained with anti-gag-PE (clone KC57, Beckman Coulter UK) for 20 mins at room temperature. Following this incubation cells were washed and fixed with BD stabilising fixative (BD Biosciences) and cells were acquired on a forward scatter versus PE plot using a 3-laser configuration LSRII flow cytometer (BD Biosciences, USA) and analysed by FlowJo (Tree Star Inc, USA).

### Statistics

Data are expressed as mean±standard deviation unless otherwise stated and were analysed by GraphPad Prism Version 5.00. When more than 2 groups were compared, statistical significance was determined using Friedman Test with Dunn’s Multiple comparison post test, or a paired t test when two groups were compared. Statistical significance was determined at p<0.05.

## Results

### Fusion of Gag to Ubiquitin Enhances Proteasomal Targeting

**Figure 1:**
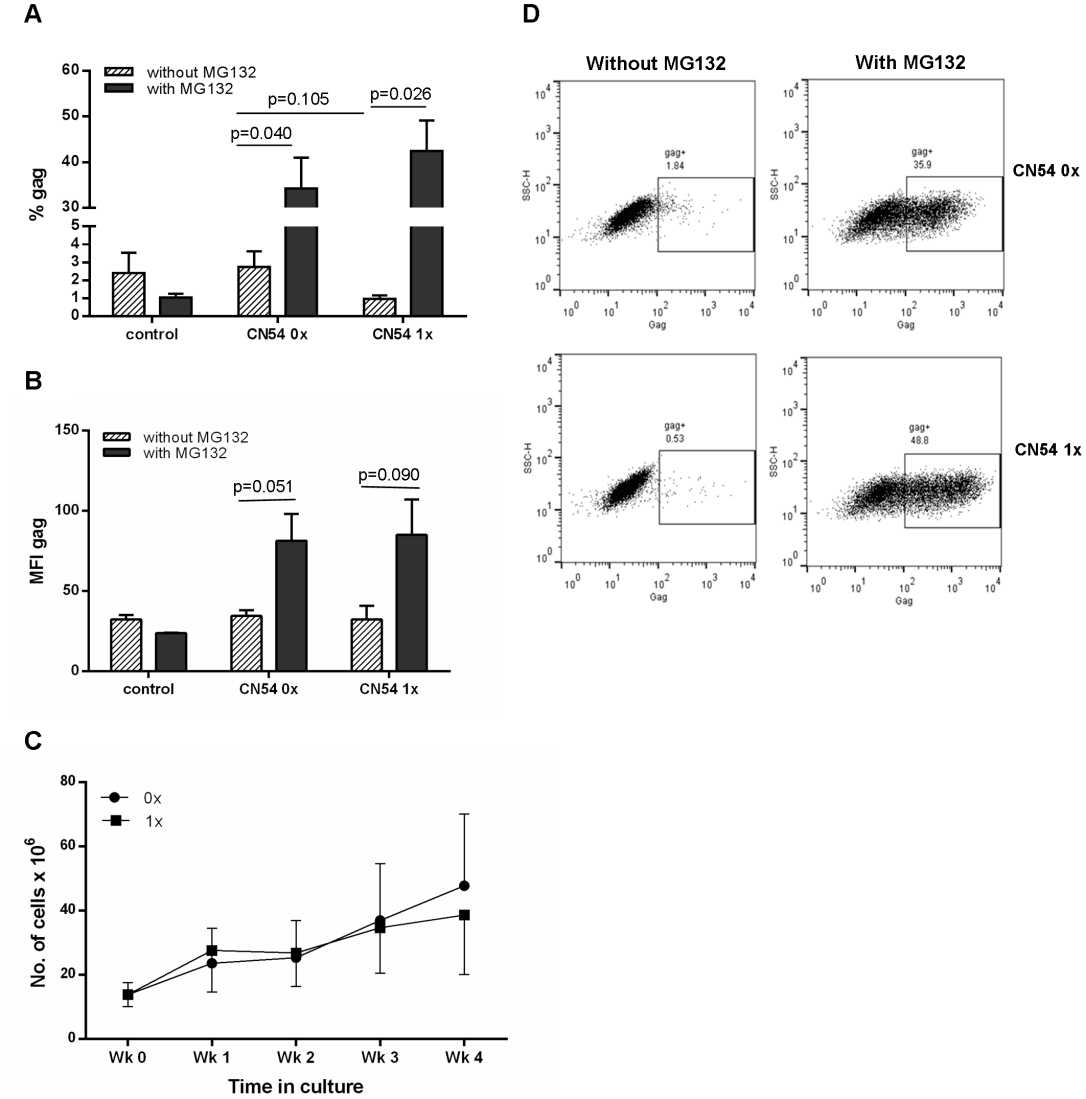
Expression of constructs and T cell expansion. A, B and D) Human moDCs were transduced with either the non-ubiquitinated (0x) or ubiquitinated (1x) HIV-1 CN54 gag constructs for 24 h or untransduced (control) and then cultured for a further 18 h in the presence (grey bar) or absence (hatched bar) of the proteasome inhibitor, MG132 (A and B). Cells were then harvested and stained intracellularly with anti-gag-PE antibody and analysed for the frequency (A) and density (B) of gag by flow cytometry. Results shown are the mean±SD for 3 donors and representative flow cytometry plots are shown in D. C) MoDCs were transduced with either the non-ubiquitinated (0x, closed circle) or ubiquitinated (1x, closed square) HIV-1 CN54 gag constructs and co-cultured with autologous naïve T cells. On a weekly basis, cells were harvested and counted by Trypan blue exclusion and co-cultured with freshly transduced moDCs. Graph shows the mean±SD of 4 donors.

To demonstrate that fusion to ubiquitin enhances targeting to the proteasome, transduced cells were incubated in the presence or absence of the proteasomal inhibitor MG132. In the absence of inhibitor the percentage of cells expressing CN54 gag ranged from 1.8 to 4.5% (mean = 2.74, SD = 1.52), for the non-ubiquitinated gag (CN54 0x) and 0.65 to 1.33% (mean = 0.96, SD = 0.34) for the ubiquitinated gag (CN54 1x) constructs (p = 0.105) (Figure 1A). In the presence of the inhibitor, expression was significantly increased in moDC transduced with either construct, with 20.7%–41.9% of CN54 0x and 33.1%–55.4% of CN54 1x transduced cells expressing gag (Figure 1A and B). However, the increase in expression was significantly more marked for the CN54 1x with a 44-fold increase compared to a 12.5 fold increase for the CN54 0x constructs. Thus fusion with ubiquitin does increase proteasomal degradation and, as noted previously [36], the overall expression level was higher for the ubiquitin fusion gene. In addition to the frequency of gag+ cells, the density of gag was determined by analysing the mean fluorescence intensity (MFI). The results in Figure 1D mirrored those observed in Figure 1A, with an increase in gag density when MG132 was added.

In addition to assessing the production of the gag protein, we also determined whether there was any difference in the viability and expansion of cells transduced with the constructs. Using Trypan blue exclusion there was no significant difference between the two constructs in terms of cell viability or the ability to expand cells (Figure 1C).

### Absence of Enhanced Responses in Ubiquitinated Protein

**Figure 2:**
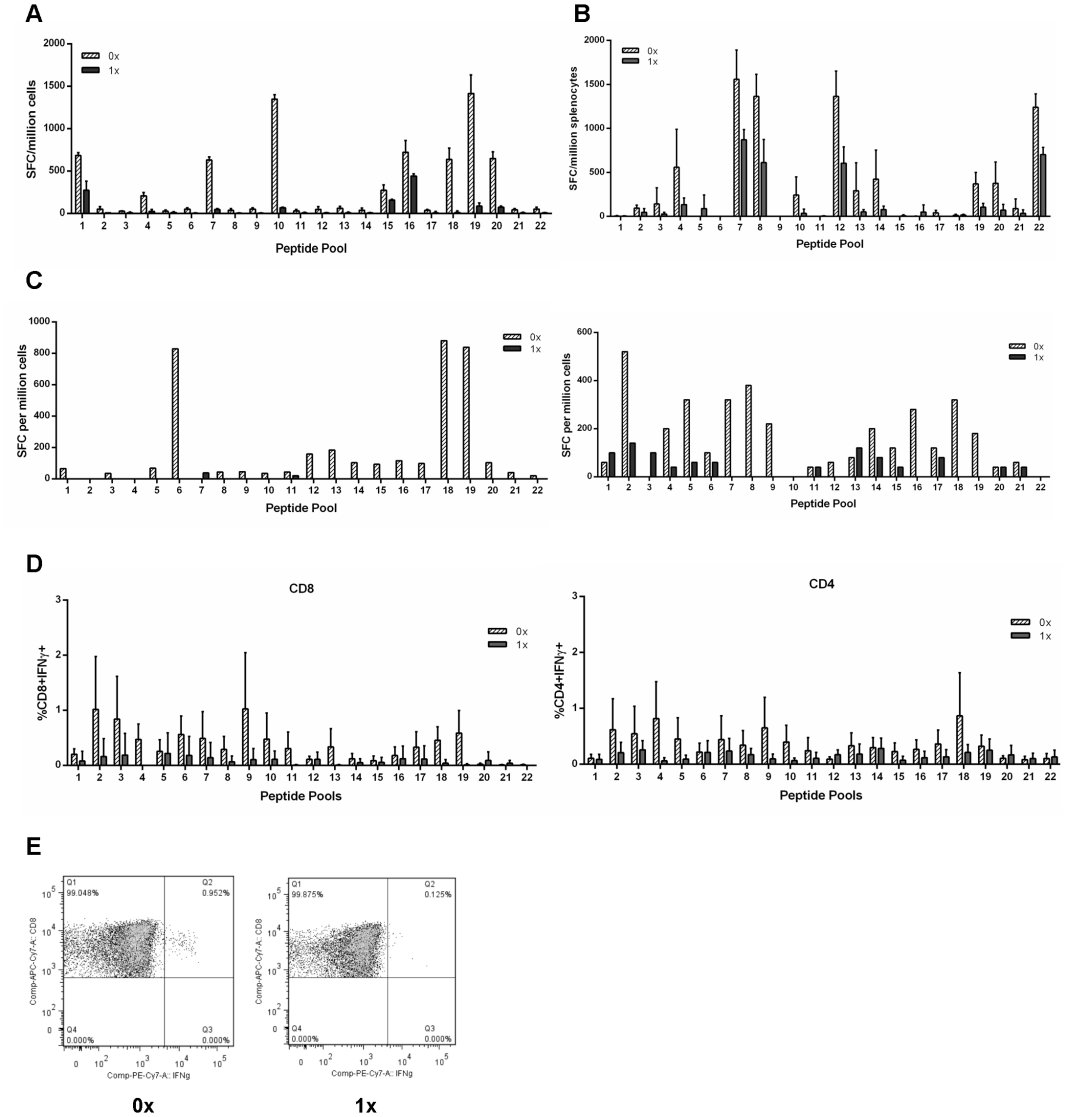
IFN gamma production by T cells treated with gag constructs. MoDCs were transduced with either the non-ubiquitinated (0x, hatched bar) or ubiquitinated (1x, grey bar) HIV-1 CN54 gag constructs and co-cultured for 7 days with autologous T cells for cells from A) HIV infected patients or C) for healthy control donors. T cells were expanded in culture for 4 weeks, harvested and restimulated with peptide pools and IFNγ production determined by ELISPOT. In addition, mice were injected with either construct and one week post injection, spleens were harvested, stimulated with peptide pools and IFNγ production was determined by ELISPOT (B). IFNγ production by T cells from healthy human controls was also determined by intracellular cytokine staining of CD8 or CD4 T cells (D and E). Graph A) represents one donor of 3± SD, B) represents 3 mice±SD and is representative of one experiment out of three, C) represents 2 individual donors out of 6, D) represents 4 donors±SD and E) is a representative CD8+ intracellular flow cytometry plot.

Previous work has shown that ubiquitination of a desired protein not only increased the expression of the protein but also the T cell responses to the protein [36], [37]. Results from Figure 1A showed that in the absence of a proteasome inhibitor, there was low expression of gag from cells treated with either the non-ubiquitinated (Ub^−^) or ubiquitinated (Ub^+^) gag constructs (1.8–4.5% and 0.65–1.33% respectively) but that in the presence of the inhibitor a high frequency of gag+ cells was detected (20.7–41.9% for Ub^−^gag vs 33.1–55.4% for Ub^+^gag), suggesting that unmodified CN54 gag is efficiently targeted to the proteasome and increased by fusion to ubiquitin. To determine whether targeting the gag protein to the proteasome translated to enhanced CTL responses, we assessed the ability of DCs transduced with the vectors to stimulated production of IFNγ by T cells from HIV-infected donors (Figure 2A). Despite the efficient proteasomal targeting of both constructs, IFNγ production was stimulated in lower numbers of T cells in cultures transduced with the Ub^+^gag constructs (Figure 2A). As we could not rule out the possibility that these findings may reflect dysfunctional cellular immunity of HIV-infected individuals we evaluated both constructs in an *in vivo* mouse model and, in a primary *in vitro* stimulation system using cells from healthy controls. Similar to the investigations with cells from HIV-infected individuals, we observed consistently lower T cell responses in the *in vivo* mouse model (Figure 2B) and in T cells stimulated with moDC transduced with the Ub^+^gag construct (Figure 2C). To evince which cell type was responsible for IFNγ production, ICS was also performed and Figures 2D and E show that both CD8 and CD4 T cells produced the pro-inflammatory cytokine and that there were fewer cytokine producing cells stimulated with Ub^+^gag constructs.

### Reduced T Cell Responses Induced by Ubiquitin Fused to a More Stable Gag Protein

**Figure 3:**
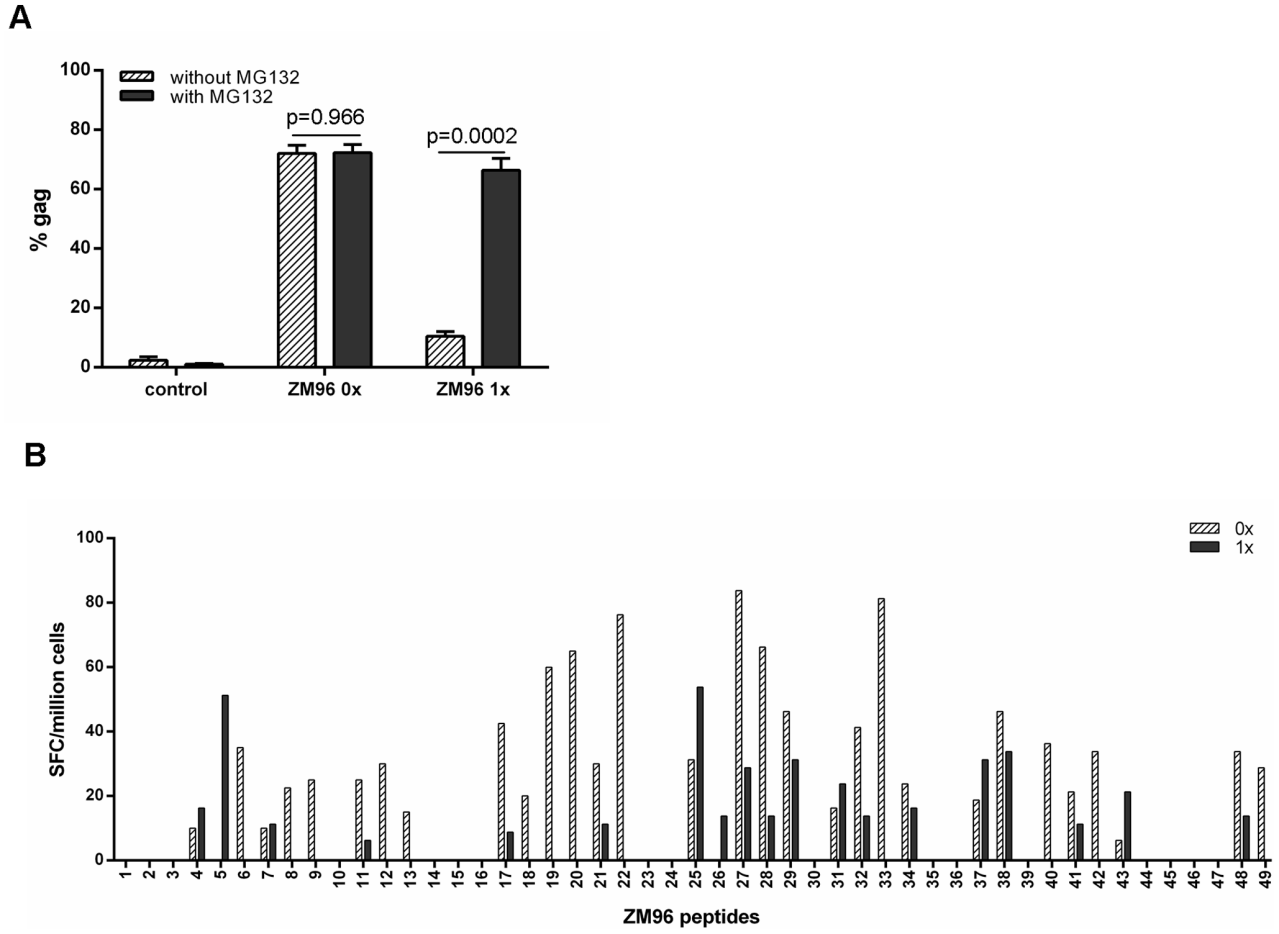
Expression of constructs and T cell responses. A) Human moDCs were transduced with either the non-ubiquitinated (0x) or ubiquitinated (1x) HIV-1 ZM96 gag constructs for 24 h or untransduced (control). Cells were then cultured for a further 18 h in the absence (hatched bar) or presence (grey bar) of the proteasome inhibitor, MG132. Cells were then harvested and stained intracellularly with anti-gag-PE antibody and analysed by flow cytometry. Results shown are mean±SD for 3 donors. B) MoDCs were transduced with either the non-ubiquitinated (0x, hatched bar) or ubiquitinated (1x, grey bar) HIV-1 ZM96 gag constructs and co-cultured with autologous naïve T cells. After 4 weeks of T cell expansion in culture, T cells were harvested and restimulated with single individual peptides and IFNγ production was determined by ELISPOT. Overall fusion with ubiquitin lowered the response although responses to 7 peptides were increased. For the 3 donors not shown only 2/49, 5/49 and 1/49 peptides gave a better response with Ub-gag. Graph represents 1 of 4 donors.

Unmodified CN54 gag is relatively unstable with most of the protein degraded by the proteasome as shown (Figure 1A). Thus enhancement of already high levels of proteasomal degradation by fusion with ubiquitin may have a negative rather than a positive effect on presentation. To determine whether the findings reflect the stability of CN54 gag, Ad5 vectors were made coding for unmodified and ubiquitin fused gag from the Clade C virus ZM96. When moDC were transduced with Ub^−^ZM96 gag vectors there was a high level of gag expression, approximately 70% (Figure 3A), with only a little increase in the presence of the proteasomal inhibitor as indicated by a slight increase in MFI on FACS analysis (data not shown). Thus ZM96 gag is inherently more stable than CN54 gag. There was a marked reduction in the level of gag expression (13%) in moDC transduced with the Ub^+^ construct which was increased to 62% (Figure 3A) in the presence of the proteasomal inhibitor with a significant increase in MFI (data not shown). When the immunogenicity of the two constructs was tested in the *in vitro* priming assay, in general lower responses were obtained with the Ub^+^ construct against individual peptides (Figure 3B). Overall fusion with ubiquitin lowered the response although responses to 7 peptides were increased. For the 3 donors not shown, only 2/49, 5/49 and 1/49 peptides gave a better response with the Ub-gag. Our CN54 gag construct lacked the myristolation site at the N terminus and to rule out the possibility that this may have accounted for the CN54 vector findings CN54 constructs with an intact myristolation site were made and tested. The results with these vectors were identical to constructs lacking the myristolation site (data not shown). Taken together with the ZM96 gag results, these findings suggest that it is the fusion of gag to ubiquitin rather than excessive degradation of gag that reduces the level of responses.

### Ubiquitination does not Inhibit Production of Immune Mediators

**Figure 4:**
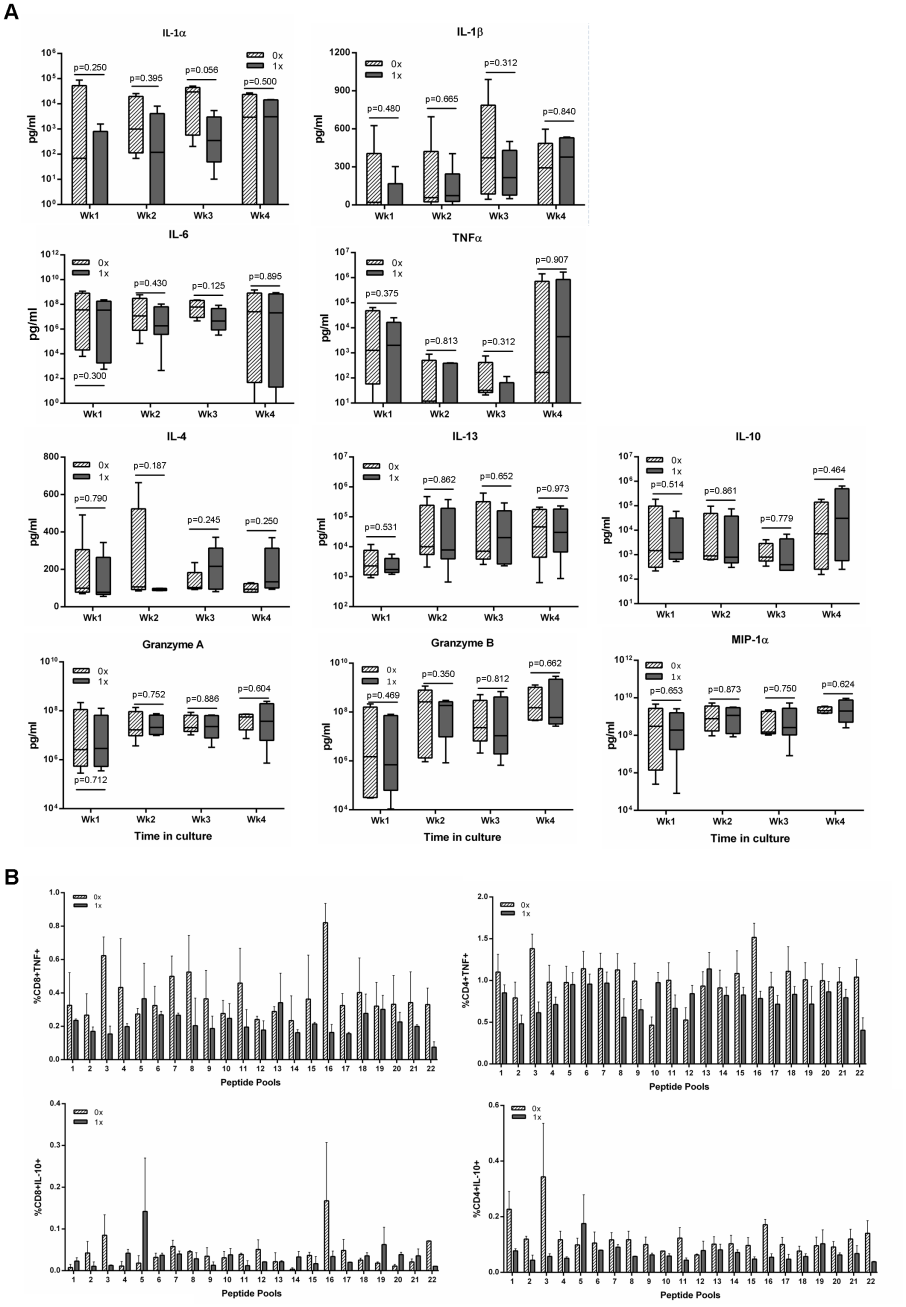
Detection of immune modulators in supernatants of cultures, or by ICS, treated with either construct. MoDCs were transduced with either the non-ubiquitinated (0x, hatched bar) or ubiquitinated (1x, grey bar) HIV-1 CN54 gag constructs and co-cultured with autologous naïve T cells. After each week, supernatants were removed and the presence of the indicated cytokines was determined by CBA and analysed by flow cytometry (A). After 4 weeks in culture, the cells were harvested and restimulated with peptide pools and the expression of TNF-α and IL-10 were determined by ICS. The box plots (A) represent the median with upper and lower quartiles, and whiskers represent the min and max values of 5 donors. Graph B show the results of 4 donors±SD.

To determine whether ubiquitination influenced the production of other immune mediators, supernatants were collected during the course of co-culture of DCs with T cells and the production of 10 immune mediators was determined. The production of IL-1α, IL-1β, MIP-1α, TNF-α, IL-6, IL-10, IL-13, IL-4, Granzyme A, and Granzyme B was detected at all time points during *in vitro* culture, although there were no significant differences in production of mediators in supernatants between the two transgene constructs (Figure 4A). To determine whether there were any differences at an epitope level, the expression of TNF-α and IL-10 were measured by ICS. The results in Figure 4B show that there were some differences at an epitope level but that overall there was no significant difference between the expression of either TNF-α or IL-10 between the two constructs.

### Ubiquitinated Gag Stimulation does not Preferentially Increase Inhibitory Molecule Expression on T Cells

**Figure 5:**
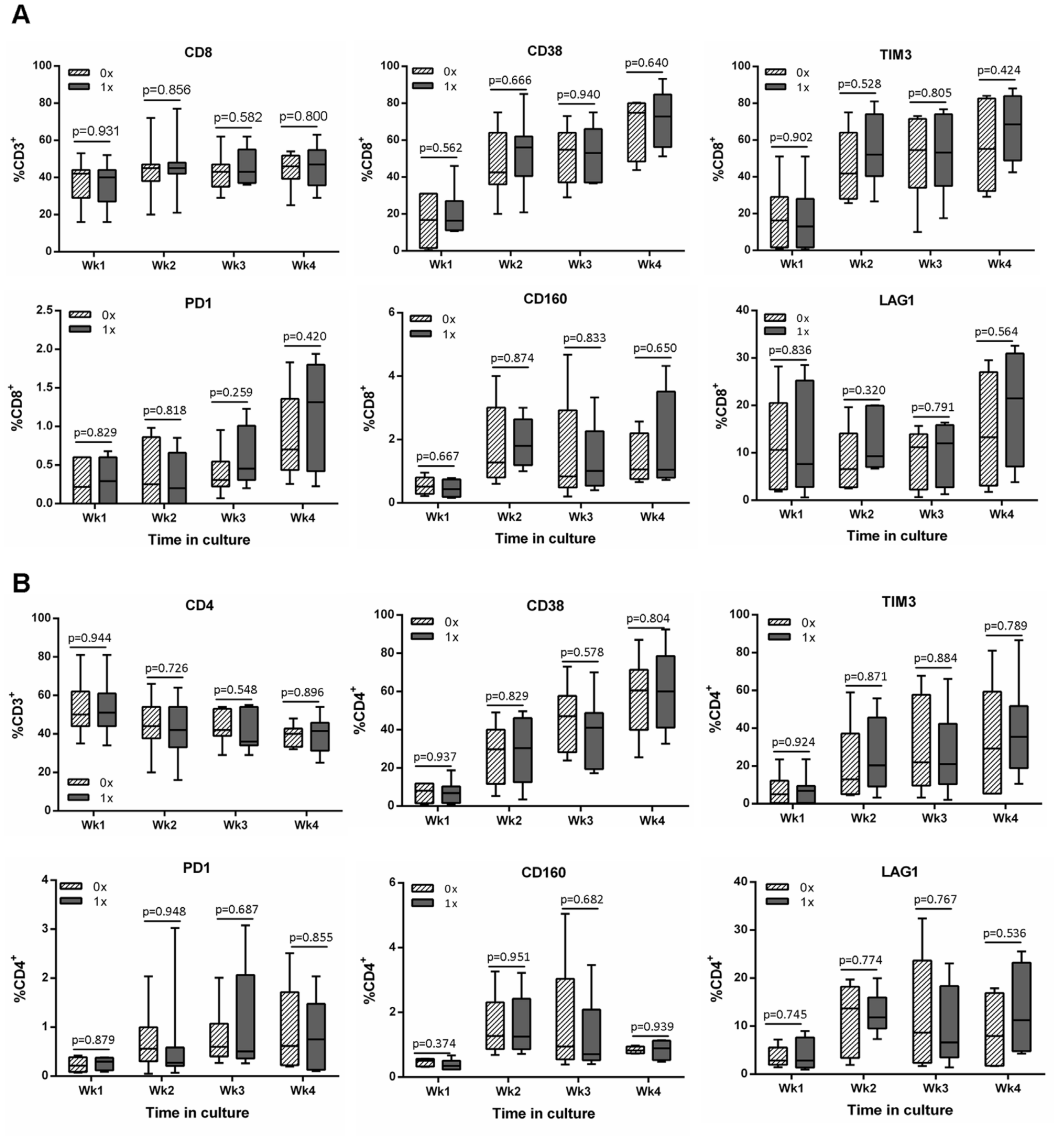
Phenotype of CD8 and CD4 T cells stimulated with either non-ubiquitinated or ubiquitinated constructs. MoDCs were transduced with either the non-ubiquitinated (0x, hatched bar) or ubiquitinated (1x, grey bar) HIV-1 CN54 gag constructs and co-cultured with autologous naïve T cells. After each subsequent week in culture, cells were removed and stained with antibodies to A) CD8 or B) CD4 and the indicated surface markers. Cells were analysed by flow cytometry, and the box plots represent the median with upper and lower quartiles, and whiskers represent the min and max values of 9 donors.

To assess whether the difference in IFNγ production between cells co-cultured with either construct was due to differences in CD8 and CD4 T cell phenotype, a panel of inhibition and activation markers was investigated (Figure 5). The frequency of CD8 T cells increased during the course of culture while there was a concomitant decrease in the frequency of CD4 T cells (Figure 5A and B respectively). All markers increased in frequency on both T cell subsets during the course of the *in vitro* culture and there was no significant difference between cells that had been stimulated with either the Ub^+^gag or Ub^−^gag constructs (Figure 5).

### Absence of Enhanced Immune Responses to Ubiquitination is a Consequence of the Gag Insert

**Figure 6:**
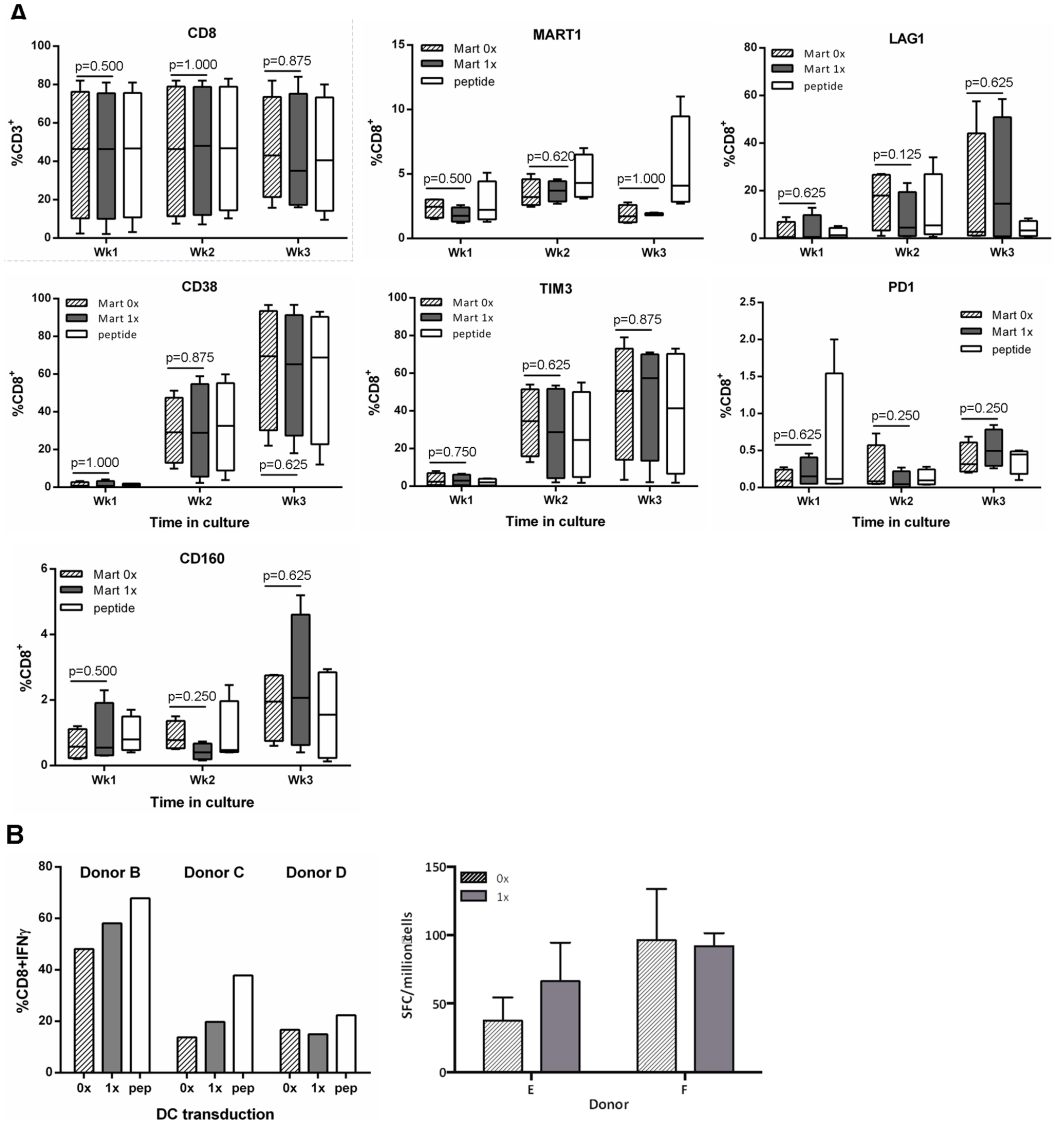
Phenotype of CD8 cells and their expression of IFN gamma from HLA-A*0201 individuals. MoDCs from HLA-A*0201 individuals were transduced with either the non-ubiquitinated (MART 0x, hatched bar) or ubiquitinated (MART 1x, grey bar) MART construct, as well as the dominant peptide ELAGIGILTV (peptide, open bar) as a positive control. DCs were then co-cultured with autologous naïve T cells and after each subsequent week in culture, T cells were harvested and stained with antibodies to the indicated surface markers (A). B) After 3 weeks of T cell expansion in culture, T cells were harvested and restimulated with the dominant peptide, ELAGIGILTV, and IFNγ production was determined by intracellular cytokine staining and ELISPOT. Cells were analysed by flow cytometry, and A) box plots represent the median with upper and lower quartiles, and whiskers represent the min and max values of 5 donors and B) shows individual donors.

To determine whether the absence of enhanced responses to the Ub^+^gag construct was due to the ubiquitination of the gag protein, another model ubiquitinated system was used to examine CD8 T cell responses. Melanin A, a melanoma protein, is recognised by an unusually high number (1∶2,500 or greater) of naïve CD8 T cells in HLA-A*02 restricted individuals [38]. The dominant peptide of MART, ELAGIGILTV, is recognised by naive CD8 T cells from HLA-A*0201 individuals and readily detectable by pentamer labelling. We identified HLA-A*0201 donors with CD8 cells that were positive for MART pentamer staining and stimulated moDCs with either a ubiquitinated MART (Ub^+^MART) or non-ubiquitinated MART (Ub^−^MART) construct. The number of pentamer positive cells generated by the vector transduced moDC increased to 2–3% over the first week of culture but did not increase in number over the following weeks (Figure 6A). Stimulation with peptide pulsed moDC expanded slightly higher numbers of MART-specific CD8 T cells and there was an increase in the expression of CD38, TIM3, PD1, CD160 and LAG1 over the course of the stimulation period (Figure 6A). Interestingly, there was no significant difference in the frequency of CD8 MART-specific T cells generated by the two constructs. To determine whether the ubiquitination of MART affected the expression of IFNγ by CD8 T cells, CD8 T cells were expanded in the presence of either Ub^+^ or Ub^−^ MART constructs and after 3 weeks, cells were harvested and expression of IFNγ was measured by intracellular cytokine staining or ELISPOT in the total CD8 population. (It was not possible to detect IFNγ in pentamer+ cells following Brefeldin A addition, as our staining protocol occurred after stimulation and were not detectable [39]). Unlike the gag transgene experiments, there was no difference in the level of IFNγ by CD8 T cells induced by the Ub^+^ or Ub^−^ transgene constructs (Figure 6B).

### Ubiquitinated Gag Suppresses Upregulation of Dendritic Cell Co-stimulatory Molecules

**Figure 7:**
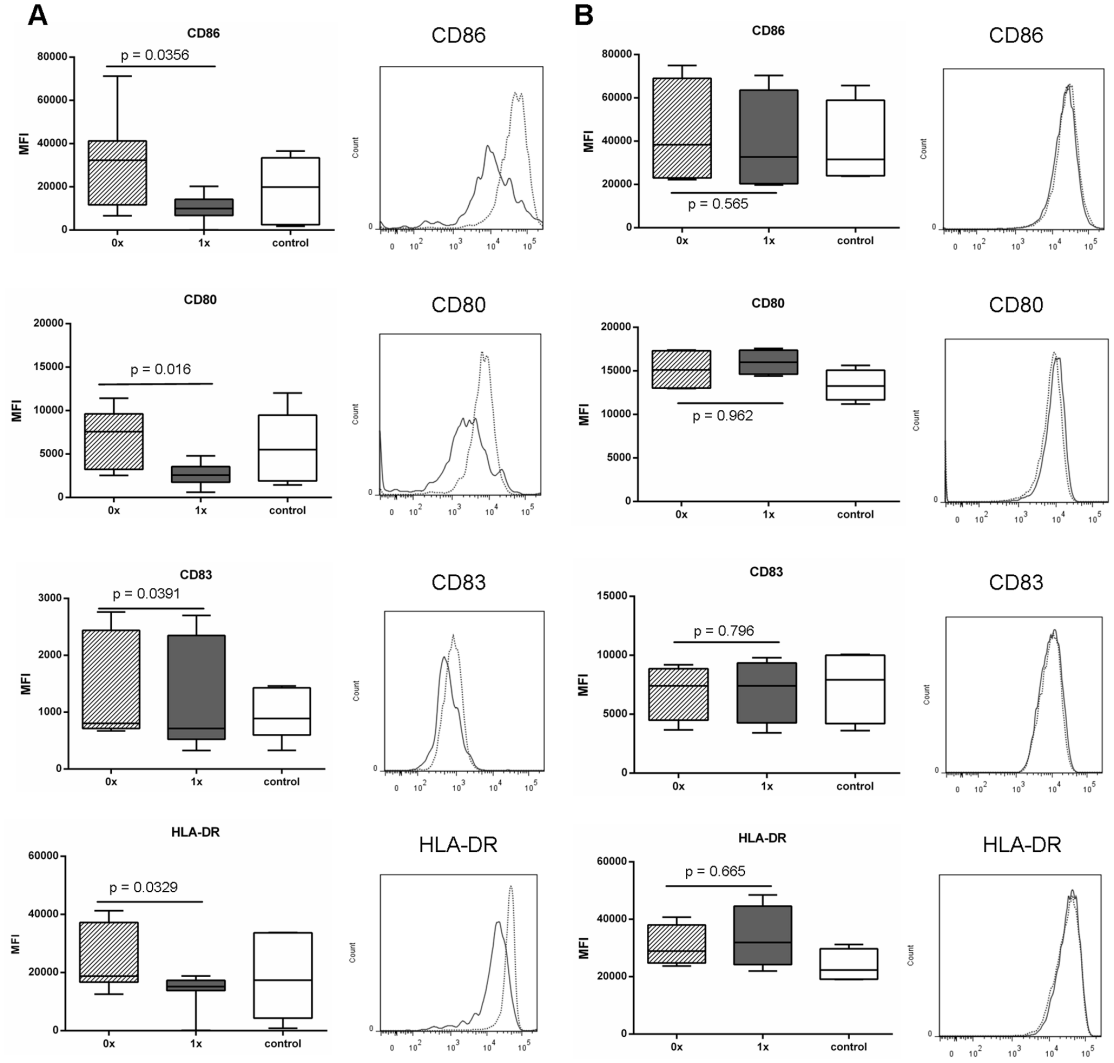
Expression of maturation markers on transduced moDCs. MoDCs were transduced with either the A) non-ubiquitinated (0x, hatched bar and dotted line) or ubiquitinated (1x, grey bar and grey line) HIV-1 CN54 gag constructs or B) non-ubiquitinated (0x, hatched bar and dotted line) or ubiquitinated (1x, grey bar and grey line) MART-1 constructs or untransduced (control, open bar). Following an overnight incubation, DCs were then matured with LPS and IFNγ for a further 24 hrs and then harvested and stained with the indicated antibodies and analysed by flow cytometry. A) represents 9 donors, and the histogram represents one donor, B) represents 4 donors and the histogram represents one donor and box plots represent the median with upper and lower quartiles, and whiskers represent the min and max values.

**Figure 8:**
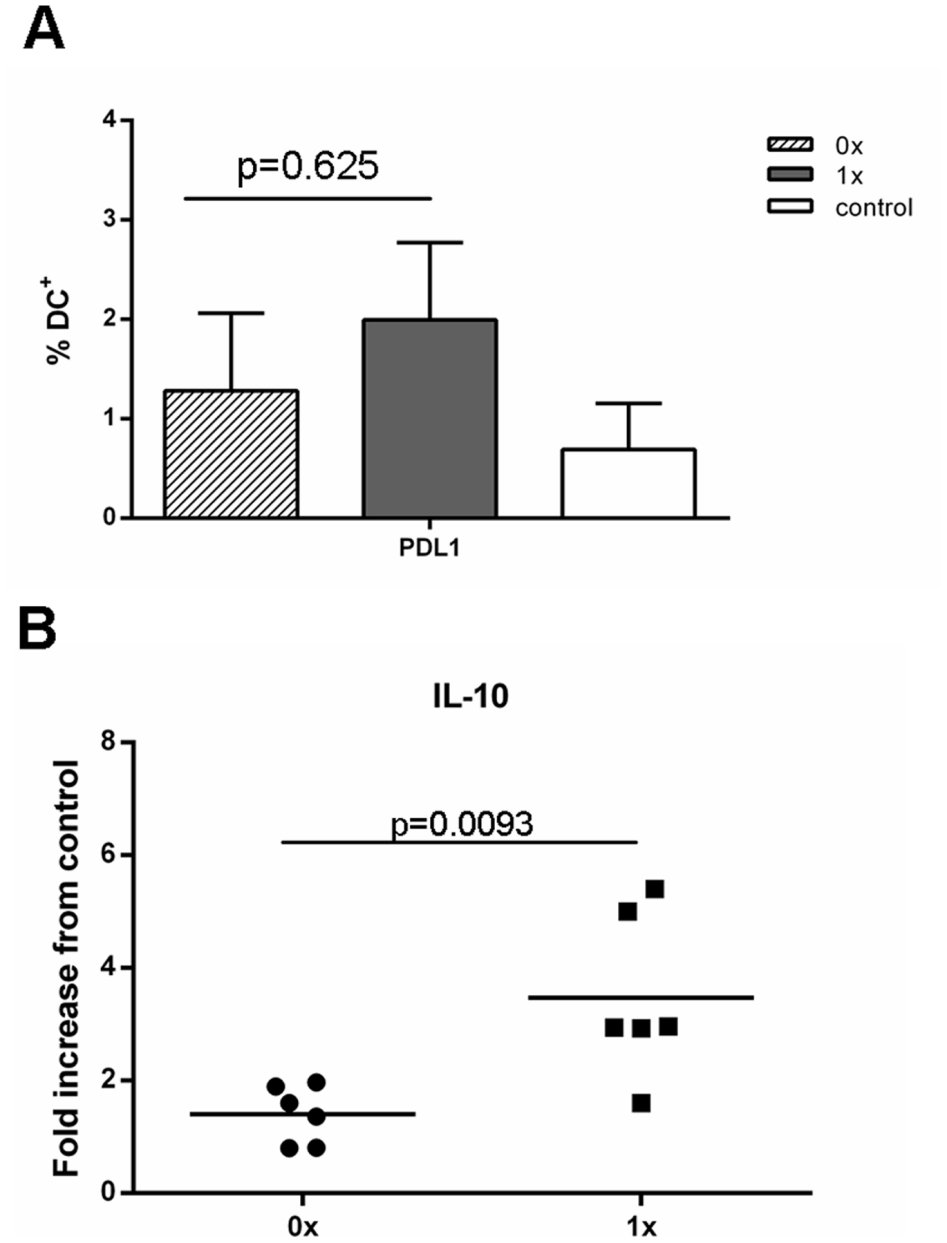
Expression of PDL1 and production of IL-10 by transduced moDCs. MoDCs were transduced with either the non-ubiquitinated (0x, hatched bar or closed circle) or ubiquitinated (1x, grey bar or closed square) HIV-1 CN54 gag constructs or untransduced (control, open bar). Following an overnight incubation, DCs were then matured with LPS and IFNγ for a further 24 hrs. Cells and supernatants were harvested and A) cells were stained with anti-PDL1 and analysed by flow cytometry and B) the production of IL-10 was determined by ELISA. Graph A) represents the mean±SD of 5 donors and graph B) represents 6 individual donors.

Results from our MART experiments showed that ubiquitination of proteins, in principle, should not reduce immune responses but as previously shown, should in some cases increase responses. Phenotypic characterisation of responding T cells showed that there were no differences between T cells stimulated with DCs transduced with either Ub^−^gag or Ub^+^gag constructs (Figure 5). Consequently, we investigated whether transduction of the moDCs with either construct affected DC maturation. Expression of CD86, CD80, CD83 and HLA-DR were significantly reduced in DCs, stimulated to mature with LPS and IFNγ, after transduction with Ub^+^gag compared to Ub^−^gag constructs (Figure 7A). There was no significant difference in viability between the transduced DCs (p = 0.888, data not shown). To elucidate whether the decrease in co-stimulatory molecules was specific to gag ubiquitination, we transduced DCs with our other constructs, Ub^−^MART and Ub^+^MART. Unlike the gag transgene constructs, there was no difference in the expression of any of the maturation markers between the Ub^+^ and Ub^−^ MART transgene constructs (Figure 7B). There was no difference in the frequency of DCs expressing the apoptotic ligand, PDL1 (Figure 8A) but a significantly higher fold increase in the amount of IL-10 in culture supernatants of DCs transduced with Ub^+^ gag (Figure 8B). This was in contrast to the expression of IL-10 by T cells which showed no difference in the percentage of cells expressing IL-10 in response to either construct (Figure 4). These results suggest that Ub^+^ gag influences T cell responses by impinging on DC maturation and is a property specifically associated with fusion of ubiquitin to HIV gag.

## Discussion

The ability to increase the magnitude and repertoire of CTLs to HIV-1 proteins has been at the forefront of HIV vaccine development for a number of years. Previous studies have shown that control of virus load is correlated not only to specific HIV proteins, namely gag, but more importantly to the breadth of the response [5] and subsequently studies have focused on increasing the magnitude and repertoire of T cell responses. The investigation into whether ubiquitination of the gag protein would yield enhanced CTL responses has been driven by previous work, which has illustrated the effectiveness of such a strategy. Modified DNA vaccines, and one report using an Ad5 deliver vector, which have incorporated a ubiquitin gene to increase targeting to the proteasome, and hence increasing the antigenic peptides to the MHC class I pathway have proved fruitful in many models [18], [40], [41]. Indeed, modification of the HIV gag protein in a murine *in vitro* model also indicated improved CTL responses [9]. However, our initial experiments investigating the expression and targeting of our transgene construct to the proteasome indicated that in the absence of ubiquitin, the CN54 gag is targeted readily to the proteasome and that the addition of ubiquitin further enhanced degradation. The question then arose as to whether the already efficient targeting of the CN54 gag to the proteasome resulted in enhanced epitope responses as a consequence of ubiquitination. Despite IFNγ responses to the non-ubiquitinated transgene construct, ubiquitination of the transgene did not enhance IFNγ production nor did it result in a broader response by CTLs, though in some donors the recognition of the odd epitope was enhanced by the ubiquitinated transgene (Figure 3B). Generally the magnitude of the response was reduced for both dominant and subdominant epitopes rather than total loss of all subdominant epitopes. This may reflect the in vitro priming system where a high ratio of antigen presenting cells (APC) to T cells is maintained throughout the culture and thus less likely to limit competition between T cells recognising dominant and subdominant epitopes on the surface of the APC. Overall, however, there was a consistently lower IFNγ response or in some cases a complete ablation of the response. The presence of enhanced responses to some epitopes and recognition of a small number of new epitopes in the ubiquitinated construct suggests that some epitopes may be recognised as a consequence of gene modification which alters antigen processing. Nevertheless the overall impact of ubiqutinating the gag gene resulted in down-regulation of IFNγ production. Despite our transgene constructs being mammalian codon optimised and fusion to ubiquitin increasing proteasomal targeting, the responses conferred by ubiquitin were reduced and seemed contradictory. However, there are some studies where the strategy of targeting the presentation pathways by ubiquitination has also failed [42], [43] suggesting that the outcome of targeting is largely dependent on the protein modified. Indeed, this proposal is supported by our study in which responses were reduced by fusion of ubiquitin to HIV gag but not to melanin. A study investigating malaria DNA vaccines also illustrated that two antigens from the same stage of the parasite life cycle responded differently to strategies designed to improve their immunogenicity [41]. The authors suggest that the difference in targeting maybe due to the inherent natural instability of the protein. In fact, our results illustrated that unmodified CN54 gag is already very efficiently targeted to the proteasome (Figure 1) suggesting that further targeting of the presentation pathway would result in little enhancement of responses and that fusion to ubiquitin suppressed responses. The view that fusion to ubiquitin reduces responses is supported by experiment with the more stable ZM96 gag where fusion to ubiquitin greatly enhances proteasomal degradation yet results in lowering of T cells responses. The high level of proteasomal targeting of unmodified CN54 gag, and presumably the class I processing pathway, may explain why the responses were higher than those observed with ZM96. The efficiency with which HIV-1 CN54 gag was targeted to the proteasome was unknown at the time of transgene construct and was based on the finding that HIV-1 ZM96 gag, which shares 90% sequence homology with HIV-1 CN54 gag, did not efficiently target to the proteasome until the gene was ubiquitinated (as shown in Figure 3). However, like the CN54 gag transgene construct, ubiquitination of the ZM96 gag did not enhance or broaden CTL responses suggesting that the reduced responses seen with the Ub^+^gag constructs did not reflect excessive proteasomal degradation. Hence, the question remains, why does ubiquitination of the CN54 gag result in diminished IFNγ responses. It is unclear why the ubiquitination of HIV gag results in less IFNγ compared to other ubiquitinated proteins, such as MART, considering that both constructs do result in the production of other immune mediators. The result suggests that ubiquitination of the HIV gag, may somehow interfere with the signalling cascade resulting in IFNγ production and requires further investigation.

Previous work on increasing immunogenicity of target antigens has determined CTL responses by assay of viral load, cytotoxicity and/or IFNγ production [18], [40], [44]. Our work has primarily determined CTL responses by IFNγ production and consequently it is possible that we have underestimated CD8+ T cell responses by limiting our readout system. However, we did measure 10 secreted immune modulators and among them were Granzyme A and B which were both produced during the course of culture. However, there was no discernible difference between the levels of Granzyme A and B or in any of the immune mediators, in supernatants from co-cultured cells stimulated with either construct. The observation of reduced IFNγ production suggests that Ub^+^ gag constructs might reduce the capacity of DCs to produce IL-12, and hence prime an optimal Th1 response. Although we did not measure the output of IL-12 by DCs transduced with either construct, we did investigate the effect of transduction on DC maturation and found that the Ub^+^ gag reduced the expression of the co-stimulatory and maturation markers, CD80, CD83, HLA-DR and CD86. Engagement of CD86 on DCs with CD28 on T cells provides T cells with the necessary co-stimulatory signals to be readily activated [45], and the down-regulation of the co-stimulatory and maturation markers on DCs as a consequence of Ub^+^gag transduction may in part explain why co-culture of these DCs with T cells results in lowered IFNγ production. Although our other measured immune mediators showed no difference in levels between constructs, it must be duly noted that this was measured in the supernatants of cultures and was therefore a cumulative level of mediators. It is possible that on a per cell basis there may have been more obvious differences, though our main readout was IFNγ production. Nevertheless, transduction of DCs with Ub^+^gag resulted in lowered expression of key surface molecules which are required for optimal priming of T cells. Interestingly, recent studies have shown that ubiquitin regulates CD86 expression and subsequently DC maturation and antigen presentation [46], [47] and that CD86 ubiquitination is facilitated by IL-10 [46]. The authors show that ubiquitination of CD86 via MARCH1, an E3 ligase, down-regulates CD86 surface expression thereby regulating immune responses. It is unknown whether MARCH1 plays a role in the down-regulation of CD86 in our system, but our results suggest that transduction of DCs with ubiquitinated gag may result in excessive IL-10 production which in turn down-regulates CD86. Indeed, we measured IL-10 at the time of transduction, and found that DCs transduced with the Ub^+^gag construct resulted in a higher IL-10 production which could result in ubiquitination of CD86. This ubiquitination could be further enhanced by the presence of exogenous ubiquitin present in the construct. Subsequent down-regulation of CD86 would therefore result in sub-optimal activation of T cells and hence reduced IFNγ production.

Our results show that despite previous indication that ubiquitination increases immune responses, due diligence must be taken when undertaking such a strategy. In fact, the choice of antigen and its inherent stability are important factors when considering ubiquitination as an approach to vaccine design. The gag protein of HIV-1 strain CN54 is unstable as indicated by our proteasomal data and therefore there may already be generation of peptides at sufficient levels to ensure maximum responses without the need to further increase proteasomal degradation through fusion to the ubiquitin gene. As shown here this did not result in an enhanced response and may have abrogated the response by impairing dendritic cell maturation. Thus, expression of key maturation markers on DCs could be used as a quick determinant to elucidating the effect of ubiquitination of a protein as a possible vaccine candidate, as well as the production of IL-10. Our results highlight the importance of not only enhancing a T cell response to HIV proteins, but also ensuring optimal dendritic cell or antigen presenting cell maturation for a successful vaccine against HIV.
